# The Impact on Relapse Rates of Switching From Oral Therapy to Long-Acting Injectable Antipsychotics in the Treatment of Psychosis: A Real-World Retrospective Study

**DOI:** 10.1192/bjo.2026.11939

**Published:** 2026-06-30

**Authors:** John Tawn, Alex Pettersen, Joseph Ingram, David Taylor, Adrian Heald

**Affiliations:** 1 Salford Royal Hospital, Salford, United Kingdom; 2 Greater Manchester Mental Health, Manchester, United Kingdom; 3 Institute of Psychiatry, London, United Kingdom

## Abstract

**Aims:**

In a community mental health setting, we studied real-world relapse rates in patients with a history of psychosis, before and after switching from oral antipsychotics to a long-acting injectable antipsychotic (LAIA). We also aimed to evaluate the relevance of metabolic/demographic factors in relapse rate.

**Methods:**

Twenty-five individuals with a past history of psychosis and variable medication concordance who were switched from oral antipsychotics to LAIAs were included in the study. Oral agents were converted to olanzapine equivalents whilst LAIA generics were converted to flupenthixol depot equivalents. The depot equivalent oral dose of olanzapine was calculated, assuming an oral bioavailability of 40%. Relapse-rates occurring over the time periods on oral therapy were compared with rates occurring following initiation of LAIAs. Relapse was defined as any psychiatric hospital admission.

A multivariable Poisson regression model including drug formulation/age/sex/smoking status/BMI were fitted with follow-up time as an offset to estimate adjusted incidence rate ratios and associations with relapse outcomes.

**Results:**

The mean±SD age of the 25 individuals was 50.6±13.9 years whilst 12 (60%) were male. Mean BMI of males/females was 28.1±9.3 kg/m^2^/ 32±6.4 kg/m^2^(higher in women) respectively. 65% of patients received either flupenthixol or zuclopenthixol depot with next most common depot being Risperidal Consta. The median (IQR) of daily oral olanzapine equivalent was 10mg (6.9 – 20.0mg)/day and after switching to depot was 16.0mg (5.4 – 40.7mg)/day. The 4-week depot equivalent dose for services users switched to depot was flupenthixol 480mg (200 – 1220mg) / 4-weeks.

Following adjustment for age/sex/smoking status/BMI, relapse rates were significantly lower during LAIA treatment compared with oral treatment (incidence rate ratio [IRR] 0.13, 95% CI 0.08–0.22, p<0.001). Thus depot antipsychotics were associated with an 87% reduction in relapse rates compared to oral therapy. Male sex/younger age/lower BMI were independently associated with higher relapse rates (Exp (beta) 1.9/0.98/0.94 respectively (p<0.05)).

**Conclusion:**

In this real-world community mental health setting, this study highlights that depot antipsychotics provide a dramatic and sustained reduction of 87% in relapse rate for individuals with a history of psychosis. These findings support early prescription of LAIAs in prevention of psychosis relapse. Further evaluation is warranted to elucidate the potential of LAIAs as a treatment option after first presentation of psychosis.

Et al:

Ruth Parkman-Eason, Salford Royal Hospital, UK

Sophie Manttan, Salford Royal Hospital, UK

Yasitha llangasekera, University of Peradeniya, Sri Lanka

